# Performance of large language models as an information resource on functional hypothalamic amenorrhea for patients and healthcare professionals

**DOI:** 10.3389/frai.2026.1788928

**Published:** 2026-06-15

**Authors:** Nancy Safwan, Jana Karam, Sarah L. Berga, Maria D. Hurtado Andrade, Kristin Cole, Stacey J. Winham, Stephanie S. Faubion, Chrisandra L. Shufelt

**Affiliations:** 1 Division of General Internal Medicine, Mayo Clinic, Jacksonville, FL, United States; 2 Center for Women’s Health, Mayo Clinic, Rochester, MN, United States; 3 Department of Obstetrics and Gynecology, University at Buffalo, Buffalo, NY, United States; 4 Women’s Health Research Center, Mayo Clinic, Rochester, MN, United States; 5 Division of Endocrinology, Diabetes, Metabolism, and Nutrition, Mayo Clinic, Jacksonville, FL, United States; 6 Department of Quantitative Health Sciences, Mayo Clinic, Rochester, MN, United States

**Keywords:** functional hypothalamic amenorrhea (FHA), healthcare professional (HCP), large language model (LLM), patient education, patient information

## Abstract

**Introduction:**

To assess and compare the accuracy, readability, and overall performance of large language models (LLMs) in answering questions about functional hypothalamic amenorrhea (FHA) for patients and healthcare professionals.

**Methods:**

A total of 11 patient-level and 15 clinician-level FHA-related questions were entered separately into four LLMs: ChatGPT 3.5 (free version), ChatGPT 4.0 (updated, paid subscription), Gemini, and OpenEvidence. OpenEvidence was used only for clinician-based questions. Responses were evaluated by three expert reviewers blinded to the LLM used who rated them as accurate and complete, accurate but incomplete, or inaccurate. A fourth reviewer resolved discordant scores. Readability for patient-level questions was assessed using the Flesch Reading Ease Score (FRES) and word count. Lower FRES scores indicate more difficult reading. Accuracy and completeness were compared using odds ratios (95% CI) with ChatGPT 3.5 as the reference model, and differences in readability were analyzed using Friedman’s test.

**Results:**

LLM performance varied across question types. For patient-level questions, ChatGPT 4.0 achieved the highest accuracy (9 of 11; 82%), followed by ChatGPT 3.5 and Gemini (each 8 of 11; 73%), with no statistically significant differences. Among clinician-level questions, OpenEvidence demonstrated perfect accuracy (15 of 15; 100%), compared with 93% for and 80% for ChatGPT 4.0 and Gemini. Completeness followed similar patterns, with OpenEvidence providing the most complete clinician responses (93%) and ChatGPT 4.0 the most complete patient-level responses (89%). Readability differed significantly among models (*p* = 0.012), with Gemini producing the most readable patient-level content (median FRES 43.5 [IQR 36.8–53.4]) compared with ChatGPT 3.5 (30.6 [16.8–48.4]) and ChatGPT 4.0 (28.8 [22.1–37.6]). Word counts did not differ significantly (*p* = 0.39).

**Discussion:**

LLMs demonstrated good overall performance in answering FHA-related questions but often provided incorrect or incomplete information. Fine tuning field-specific data, engineered prompts, and obtaining human-in-the-loop feedback may help improve the accuracy of these models.

## Introduction

Large language models (LLMs), a form of generative artificial intelligence (AI), are rapidly emerging as transformative tools in healthcare, offering immediate access to medical information for both patients and healthcare professionals (Ayers et al., 2023). Platforms such as ChatGPT (OpenAI, 2025) and Gemini (previously known as Google Bard) (Google AI, 2025), have gained widespread attention for their ability to process vast amounts of data, synthesize complex concepts, and engage users through interactive dialogs. Recent research highlights the growing integration of LLMs in medical practice, incorporating them as valuable resources for patients seeking information and for clinicians caring for patients (Kung et al., 2023). Their broad public accessibility offers an opportunity to shift beyond traditional search tools such as online forums, textbooks, and journal articles to these more advanced digital models (Singhal et al., 2023). However, concerns remain about their ability to consistently deliver accurate, contextually relevant, and clinically reliable information (Singhal et al., 2023).

Functional hypothalamic amenorrhea (FHA) is a form of ovulatory dysfunction that affects reproductive-aged women as a result of psychosocial stress, disordered or restrictive eating, and/or excessive exercise. It is estimated that FHA occurs in nearly 1 million women between the ages of 15 and 44 in the U.S. (U.S. Census Bureau, 2023). While it clinically presents as amenorrhea, the underlying neuroendocrine disruption extends well beyond the hypothalamic–pituitary-ovarian axis, involving other pathways that result in upregulation of cortisol, functional hypothyroidism, and altered metabolism (Saadedine et al., 2023). FHA is associated with adverse long-term health consequences, including infertility, reduced bone mineral density, mood dysregulation, and vascular dysfunction (Shufelt et al., 2017; Shufelt et al., 2023). Despite its clinical relevance, many individuals affected by FHA experience delays in diagnosis as it is a diagnosis of exclusion, and many clinicians may not include it in their differential diagnosis or know how to arrive at a diagnosis (Saadedine et al., 2023; Gordon et al., 2017).

Although existing research suggests that LLMs can provide accurate information for certain diseases and common medical questions (Ayers et al., 2023; Cohen et al., 2025; Yeo et al., 2023), it is important to recognize that their responses are generated based on patterns learned from large datasets, including publicly available medical literature, guidelines, and web content. However, like many rare or less commonly studied conditions, FHA suffers from a limited body of research, which may limit the depth and accuracy of information available to LLMs. This underscores the importance of evaluating LLM performance not only for common medical conditions, but also for those that are less prevalent yet clinically significant. Given the complex and multifactorial etiology of FHA, it is unclear whether LLMs, when used as a complementary tool in healthcare, can effectively deliver accurate, comprehensive, and accessible information.

This study aimed to assess the performance of four LLMs in addressing FHA-related questions. The primary outcome was the accuracy of LLM-generated responses as evaluated by expert reviewers. Secondary outcomes included the completeness of responses and the readability of patient-level content.

## Materials and methods

### Study design and question development

A total of 26 questions related to FHA were entered into the LLMs, comprising 11 patient-level questions and 15 clinician-level questions. Two expert clinicians on the topic (SSF and CLS) and two clinical research fellows (NS and JK) developed the question set. Questions reflected frequently encountered clinical and patient concerns and were aligned with evidence-based guidelines to ensure both relevance and content validity. Topics covered included FHA definition, diagnosis, management, and treatment options (a full list is provided in Supplementary Table S1).

Because FHA is a specialized condition with limited publicly available patient-facing content, we prioritized clinically representative questions rather than exhaustive coverage of all possible FHA scenarios. We acknowledge that this sample may not capture the full spectrum of FHA-related concerns.

### LLM response collection

Each question was entered as a separate, independent prompt into the four LLMs: (i) ChatGPT 3.5 (unpaid, free version, Open AI, Inc., San Francisco, CA); (ii) ChatGPT 4.0 (updated, paid subscription, Open AI, Inc., San Francisco, CA) (OpenAI, 2025); (iii) Gemini (formerly Google Bard, Google, LLC, Mountain View, CA) (Google AI, 2025); and (iv) OpenEvidence (Mayo Clinic Platform Accelerate Program, Rochester, MN) (OpenEvidence, 2025). OpenEvidence, a platform designed for healthcare professionals, was evaluated only for clinician-level questions, as access requires using clinician-specific credentials. Sample responses from each LLM are presented in Table 1. All questions were entered into LLMs by a single researcher (NS) to ensure consistent phrasing. Each question was entered only once per LLM, and the response generated was saved and subsequently reviewed by all expert reviewers. This ensured that all reviewers assessed the same output for each question across models and reflected a real-world user interaction. However, because LLM outputs may vary across repeated prompts, single-response evaluation may not capture the full range of possible outputs.

**Table 1:** Sample responses from the 4 large language models to clinician-level question: “How common is FHA?”

Large language model	Sample response to clinician-level question: “How common is FHA?”
ChatGPT-3.5	Functional hypothalamic amenorrhea (FHA) accounts for about 20–35% of secondary amenorrhea cases in women of reproductive age. It results from disruptions in the hypothalamic–pituitary-ovarian axis due to factors like: Low energy availability (e.g., eating disorders, restrictive diets). Excessive exercise, especially in athletes. Psychological stress or chronic stress. Low body weight or significant weight loss. FHA is common among athletes, those with disordered eating, and individuals under high stress. It is reversible with lifestyle adjustments, including improved nutrition, reduced stress, and moderated exercise.
Gemini	Functional hypothalamic amenorrhea (FHA) is a common cause of secondary amenorrhea, affecting approximately 30% of women of reproductive age. It is estimated to affect 1.62 million women in the US and 17.4 million women worldwide. FHA is more common in women who: Are athletes or engage in excessive exercise Have a history of eating disorders or disordered eating Are underweight or have experienced significant weight loss Are experiencing high levels of stress It’s important to note that FHA is a reversible condition if the underlying causes are addressed.
ChatGPT-4	Functional hypothalamic amenorrhea (FHA) is a form of chronic anovulation not attributable to organic or structural disorders. It is fairly common among women of reproductive age, particularly in populations undergoing significant stress, weight loss, or engaging in high levels of physical activity. FHA affects roughly 3% of women overall, but the prevalence can vary significantly in certain populations. For instance, among athletes, particularly those in sports that emphasize leanness or weight control like gymnastics or ballet, the prevalence can be as high as 60%. Similarly, women experiencing psychological stress or those with eating disorders like anorexia nervosa may also exhibit higher rates of FHA. The condition results from disruptions in the hypothalamic–pituitary-ovarian axis, often triggered by physical or psychological stress, excessive weight loss, or excessive exercise. These stressors can lead to reduced gonadotropin-releasing hormone (GnRH) secretion, which in turn decreases the production of the hormones necessary for ovulation.
OpenEvidence	Functional hypothalamic amenorrhea (FHA) is a common cause of secondary amenorrhea, accounting for approximately 20–35% of cases. This condition is characterized by the disruption of gonadotropin-releasing hormone (GnRH) pulsatile secretion due to factors such as psychosocial stress, disordered eating, and excessive exercise. The American College of Obstetricians and Gynecologists notes that FHA is particularly prevalent among slender female athletes, with 16 to 47% of this population at risk due to disordered eating and high levels of physical activity. In summary, FHA is responsible for a significant proportion of secondary amenorrhea cases, with estimates ranging from 20 to 35% in the general population and higher prevalence in specific at-risk groups such as athletes.

### Response evaluation and scoring

Responses to both patient-level and clinician-level questions were assessed by expert reviewers. Four expert reviewers (SSF, CLS, MDHA, SLB) evaluated LLM responses; three reviewers (SSF, CLS, MDHA) independently rated each response, and discrepancies were adjudicated by a fourth reviewer (SLB). Both the reviewers and adjudicator were blinded to the identity of the LLMs and to each other’s evaluations. LLM responses were evaluated for concordance with the Endocrine Society guidelines and alignment with expert opinion (Gordon et al., 2017). Responses were rated as follows:

- Accurate and complete if they contained guideline-concordant information and addressed the key clinical elements expected for the question.
- Accurate but incomplete if they were factually correct but omitted clinically important details.
- Inaccurate if they included incorrect, misleading, unsupported, or potentially unsafe information.

To illustrate how LLM responses were evaluated, a representative response rated as accurate and complete, one rated as accurate but incomplete, and one rated as inaccurate by all reviewers are provided in Supplementary Table S2. Reviewers also screened for hallucinations, defined as factually incorrect or fabricated statements not supported by clinical guidelines or existing evidence. Institutional Review Board approval was not required for this study.

### Readability and word count

For patient-level questions, the readability of the responses was assessed using a validated readability assessment tool, the Flesch Reading Ease Score (FRES) and word count. The FRES scale ranges from 0 to 100 and considers average sentence length and the number of syllables per word. FRES scores correspond to U.S. school grade levels, with lower scores indicating more difficult-to-read text that requires a higher educational requirement, whereas higher scores reflect easier-to-read content. For example, a score between 90 and 100 is typically understandable by a 5th-grade student, while scores of 80–90 align with a 6th-grade reading level. Scores from 70–80 to 60–70 correspond to 7th and 8th–9th grade levels, respectively. More complex texts scoring between 50 and 60 are suited for 10th to 12th graders, while scores between 30–50 and below 30 reflect college and college graduate reading levels, respectively (Flesch, 2025). Word count was measured using Microsoft Word’s automated word counter. It was included as a measure of readability, as it is well established that readability depends inversely on word and sentence length, meaning that longer responses are generally more difficult to read and more complex to understand (Matthews and Folivi, 2023). FRES scores and word counts were summarized using medians with interquartile ranges (IQR) and means with standard deviations (SD), respectively.

### Statistical analysis

Categorical outcomes (accuracy and completeness) were summarized as counts and percentages. Accuracy (primary outcome) was compared across models using repeated measures logistic regression, and was summarized using odds ratios (95% CI) with ChatGPT 3.5 as the reference. Completeness (secondary outcome) was reported descriptively as the proportion of accurate responses that were also rated complete. Differences in readability metrics across the LLMs were compared across the models using Friedman’s test for non-parametric repeated measures.

## Results

### Accuracy (primary outcome)

The performance of LLMs varied across both patient- and clinician-level questions. For the 11 patient-level questions, ChatGPT 4.0 achieved the highest accuracy (9 of 11; 82%), followed by ChatGPT 3.5 (8 of 11; 73%) and Gemini (8 of 11; 73%). Comparative analysis demonstrated that, relative to ChatGPT 3.5, neither Gemini (OR 1.00, 95% CI 0.19–5.36; *p* > 0.99) nor ChatGPT 4.0 (OR 2.22, 95% CI 0.38–13.18; *p* = 0.38) showed significantly different odds of producing accurate responses.

Among the 15 clinician-level questions, OpenEvidence achieved the highest accuracy (15 of 15; 100%), followed by ChatGPT 3.5 (14 of 15; 93%), ChatGPT 4.0 (12 of 15; 80%), and Gemini (12 of 15; 80%). Compared with ChatGPT 3.5, the odds of producing accurate responses were not significantly different for Gemini (OR 0.37, 95% CI 0.04–3.09), ChatGPT 4.0 (OR 0.37, 95% CI 0.04–3.09), or OpenEvidence (OR 3.21, 95% CI 0.11–94.87).

### Completeness (secondary outcome)

Among accurate responses (i.e., those rated as either accurate but incomplete or accurate and complete) to patient-level questions, ChatGPT 4.0 produced the highest proportion of complete answers, with 8 of 9 accurate responses (89%) rated as complete. ChatGPT 3.5 and Gemini followed, each with 6 of 8 (75%) accurate responses rated as complete. Compared with ChatGPT 3.5, neither Gemini (OR 1.00, 95% CI 0.12–8.73) nor ChatGPT 4.0 (OR 0.45, 95% CI 0.04–5.84) demonstrated significantly different odds of producing a complete response.

For clinician-level questions, OpenEvidence achieved the highest proportion of complete responses (14 of 15; 93%), followed by ChatGPT 3.5 (10 of 15; 67%), ChatGPT 4.0 (7 of 15; 47%), and Gemini (10 of 15; 67%). Odds ratios for completeness relative to ChatGPT 3.5 did not demonstrate significant differences across models (ChatGPT 4.0: OR 0.44, 95% CI 0.10–1.92; Gemini: OR 1.00, 95% CI 0.22–4.56; OpenEvidence: OR 7.00, 95% CI 0.71–69.48).

### Readability (secondary outcome)

Significant differences were found in FRES scores for patient-level responses (*p* = 0.012), with Gemini scoring the highest at 43.5 (36.8, 53.4), followed by ChatGPT 3.5 at 30.6 (16.8, 48.4), and ChatGPT 4.0 scoring the lowest at 28.8 (22.1, 37.6). There were no significant differences in word count across models (*p* = 0.39), with mean word counts of 215.4 ± 74.8 for ChatGPT 3.5, 248.6 ± 69.3 for Gemini, and 263.2 ± 81.5 for ChatGPT 4.0. These FRES scores correspond to approximately college-level reading, above commonly recommended levels for patient materials.

## Discussion

In this study, LLMs demonstrated good overall performance (accuracy >70%) when addressing questions related to FHA, although no model significantly outperformed the others. ChatGPT 4.0 achieved the highest accuracy on patient-level questions and OpenEvidence attained perfect accuracy (100%) for clinician-level questions, with no response rated as incorrect. Despite its higher accuracy, the significantly lower FRES for ChatGPT 4.0 suggests that its responses were written at a higher complexity level, making them more suited for readers with advanced knowledge, while being difficult for the average patient to read or understand. In contrast, Gemini produced slightly less accurate but more readable responses, potentially making its content easier for patients to understand.

To our knowledge, this is the first study to specifically evaluate LLM-generated content on FHA. Our findings are consistent with prior studies evaluating LLM performance in other clinical domains, although direct comparisons should be interpreted cautiously given differences in study design, question sets, and clinical context. Previous research has shown mixed results regarding the usefulness of LLMs in addressing patient questions, with studies on subjects such as bariatric surgery, endometriosis, and heart rhythm disorders finding LLMs to be accurate and comprehensive with correct responses often exceeding 80% (Cohen et al., 2025; Samaan et al., 2023; Van Bulck and Moons, 2024; Kassar et al., 2024). Similarly, a cross-sectional study of 195 patient questions in the field of Internal Medicine found that LLM-generated responses were preferred over physician responses by an expert panel in 78.6% of cases, largely because they were rated as higher quality and more empathetic (Ayers et al., 2023). Moreover, although AI tools can support patient and clinician education, several studies indicate that their responses frequently lack key clinical details and do not consistently align with established guidelines, often omitting essential information (Cohen et al., 2025). Importantly, because our analysis was limited to FHA, a relatively specialized and under-recognized condition, these findings should not be generalized to broader clinical use of LLMs without further validation across diverse medical topics.

The considerable variability in LLM performance in this study is particularly notable given the growing use of AI models by both patients and clinicians. Management of FHA requires careful consideration of diverse underlying etiologies, individual patient characteristics, and the limited evidence base due to a scarcity of literature and lack of research on the topic. This complexity presents a significant challenge for any algorithm, including AI-based LLMs, as demonstrated by our findings. Importantly, FHA is strongly influenced by psychosocial factors, including stress, eating behaviors, and exercise patterns, which require nuanced and individualized counseling. These aspects may be particularly difficult for LLMs to address. For instance, a recent study evaluating ChatGPT 3.5 and 4.0 in low back pain patient education found that, despite strong performance in diagnostic and treatment-related questions, both models demonstrated significantly lower performance when addressing psychosocial concerns (Tabanli and Demirkiran, 2025). This limitation is especially relevant in FHA, where effective management depends not only on clinical knowledge but also on addressing behavioral and psychosocial contributors to disease. In our study, responses to questions regarding appropriate assessment or treatment strategies for FHA were often incomplete or incorrect. This may be due to the evolving and limited evidence surrounding FHA, as well as the essential role of clinical judgment that cannot be fully replicated by AI models. Among patient-level questions, inaccuracies and incomplete answers were most common in areas related to treatment decision-making and evaluation of long-term health consequences.

While these LLMs gather information from the Internet, including medical textbooks and scientific literature, the content they provide may not be consistently accurate or comprehensive. A likely explanation is that LLMs are trained on data sources that may not incorporate the most up-to-date clinical guidelines and recommendations. Moreover, LLMs generate responses in real-time with no integrated mechanism for fact-checking or proofreading, increasing the risk of delivering incomplete or inaccurate information (DeVerna et al., 2024). Consistent with our results, another study in hepatology reported frequent inaccurate and incomplete answers in LLM-generated responses to patient questions about cirrhosis and hepatocellular carcinoma (Yeo et al., 2023). With increasing pressure on clinician time and the ease with which patients access medical information online, interest in using LLMs to address common health-related questions continues to grow. While LLMs offer convenient and interactive access to medical information, their use for specialized conditions such as FHA must be approached with caution, particularly given the limited understanding of the condition and the ongoing lack of clarity in its definition, diagnostic criteria, and treatment guidelines. Patients and clinicians should be aware of the potential for incomplete or inaccurate information, particularly when relying on LLMs that are not explicitly designed for medical use. Within the context of FHA, these findings highlight the need for reliable, evidence-based resources to be more widely accessible online. It also reinforces that LLMs should not be used in isolation to guide important healthcare decisions. Patients are encouraged to consult with healthcare professionals who have expertise in this specific condition, as they are best equipped to detect and correct misinformation, recommend appropriate assessments, and provide evidence-based treatment options.

This study also highlights the importance of balancing accuracy and readability in clinical applications of LLMs designed to support patient understanding. The American Medical Association recommends that patient education materials be written at or below a sixth grade reading level (Weiss, 2007). However, all LLMs evaluated in our study generated responses that exceeded this recommendation, requiring more advanced reading skills. This aligns with prior research showing that LLM-generated educational content often exceeds the recommended reading levels, potentially limiting patient understanding and comprehension (Razdan et al., 2024; Kianian et al., 2024). Customizing patient education materials based on individuals’ health literacy can enhance adherence and may lead to better clinical outcomes (Miller, 2016).

For clinician-level questions, the notably better performance of OpenEvidence with no responses rated as incorrect suggests that LLMs tailored for healthcare professionals, with embedded evidence-based resources, may be better suited for complex medical queries to support clinical decision-making. OpenEvidence features multimedia content and scientific evidence from sources such as the New England Journal of Medicine, which may explain these findings (OpenEvidence, 2025). In contrast, the lower accuracy rates observed with ChatGPT-3.5, ChatGPT-4, and Gemini highlight a key limitation of general-purpose LLMs, their lack of consistent reliability when addressing complex, less well-recognized conditions like FHA, which require nuanced clinical understanding and specialized knowledge. Unlike OpenEvidence, which draws from peer-reviewed medical literature, general models are more likely to rely on widely available but potentially inaccurate or even outdated information, resulting in occasionally misleading responses. As LLMs continue to evolve, enhancing their clinical utility will depend on several key strategies: fine-tuning with field-specific, up-to-date datasets, designing engineered prompts that guide the model toward clinically relevant and evidence-based answers, incorporating human-in-the-loop approaches to provide expert oversight and iterative correction, and integrating real-time access to validated medical databases and guidelines (Yang et al., 2023). Together, these improvements will help bridge the gap between general language modeling and the precise demands of specialized medical decision-making.

While this study offers valuable insight into how LLM responses differ in addressing common patient- and clinician-level questions about FHA, several limitations should be considered. First, the scope of our evaluation was limited to FHA-specific questions, which limits the generalizability of our findings to other clinical topics. Second, although our reviewer panel included experts with substantial knowledge about FHA, assessment of LLM responses inherently involves some subjectivity. However, blinding of reviewers and the use of an adjudicator mitigated this potential bias. In addition, although responses were evaluated for concordance with the Endocrine Society Clinical Practice Guideline, we did not employ a standardized quantitative guideline-based scoring system or external objective benchmark, which may limit the reproducibility of our assessment. Third, the dynamic and evolving nature of LLMs means that model performance may change over time as updates and refinements are made; our findings represent a snapshot of model capabilities at the time of study. Finally, we did not assess user experience, response latency, or other usability factors that may also influence real-world application of these tools.

Importantly, despite relatively high accuracy rates, the presence of incomplete and occasionally incorrect responses, particularly in clinically relevant areas such as diagnosis and management, raises concerns about whether this level of performance is sufficient for safe, independent clinical use. Even a small proportion of inaccurate information may have meaningful clinical implications in practice.

While this study provides insight into how LLMs perform when addressing FHA-related questions, its findings are specific to FHA and should be interpreted within this context. Broader conclusions regarding the role of LLMs in healthcare require evaluation across a wider range of clinical conditions, question types, and real-world use settings. Future studies incorporating standardized scoring frameworks and objective knowledge benchmarks are needed to better define thresholds for clinical adequacy and safety. Until such data are available, the use of LLMs for medical information, particularly in complex conditions such as FHA, should remain cautious and guided by clinician oversight.

## Data Availability

The original contributions presented in the study are included in the article/Supplementary material, further inquiries can be directed to the corresponding author.
