# Supplementary material for: Performance of large language models as an information resource on functional hypothalamic amenorrhea for patients and healthcare professionals
Source: Front Artif Intell. 2026 Jun 15;9:1788928. doi: 10.3389/frai.2026.1788928 (PMC13311067; doi:10.3389/frai.2026.1788928)
Supplement: Supplementary file 1 [file Table_1.docx]

**Supplementary Table 1: A full list of patient- and clinician-level questions used for the different Large Language Models.**

**Part 1: Questions from patients**

1. I know I am not pregnant but why have I not had my period in 3 months?
2. What is functional hypothalamic amenorrhea?
3. Can stress make me miss my period?
4. Could my diet be affecting my period?
5. Does exercising too much affect my period?
6. Can missing my period due to FHA impact my ability to become pregnant?
7. What type of doctor should I see if my period has stopped?
8. What blood tests should I get if my period stops?
9. How do I get my period back if I have FHA?
10. How long will it take to get my period back if I have FHA?
11. Should I start on a birth control pill to get my period back if I have FHA?

**Part 2: Questions from providers**

1. What diagnostic tests should I order if I suspect my patient has FHA?
2. What conditions would need to be ruled out first before diagnosing my patient with FHA?
3. How common is FHA?
4. What are the recommendations for protection of bone health in patients with FHA?
5. Should I prescribe birth control pills for my patients with FHA?
6. Aside from low estrogen, are there other hormonal changes seen in patients with FHA?
7. Are there long-term health risks associated with FHA?
8. How can FHA be distinguished from PCOS in patients with amenorrhea?
9. I have a patient with the following age 27 years, last period from June 2021, BMI of 22.5 and estradiol 24.4 pg/ml, LH 4.58, FSH 3.55, testosterone 13.9, free testosterone 0.188, TSH 0.96, prolactin 2.89, hCG negative, what do you think is the diagnosis?
10. How do I treat my patient with functional hypothalamic amenorrhea?
11. How often should I see my patient with FHA?
12. Are there specialists or other health care providers I should send my patient with FHA to?
13. What are stress management strategies for patients with FHA to support recovery?
14. What specific nutritional interventions are recommended for patients with FHA to support recovery?
15. What guidance can be provided for FHA patients regarding exercise modifications to support recovery?
