# Supplementary material for: Performance of large language models as an information resource on functional hypothalamic amenorrhea for patients and healthcare professionals
Source: Front Artif Intell. 2026 Jun 15;9:1788928. doi: 10.3389/frai.2026.1788928 (PMC13311067; doi:10.3389/frai.2026.1788928)
Supplement: Supplementary file 2 [file Table_2.docx]

| Question | Full LLM Response | Reviewers’ Comment |
| --- | --- | --- |
| Q5 (Patient-level) Does exercising too much affect my period?  LLM: Gemini | Yes, exercising too much can affect your period. In fact, it's one of the most common causes of irregular or missed periods in women who are physically active. This condition is called functional hypothalamic amenorrhea (FHA).  Here's how it happens:  **Energy Imbalance:** When you exercise a lot, your body uses more energy than it takes in. This can lead to a state of energy imbalance, where your body doesn't have enough energy to support all of its functions, including menstruation.  **Hormonal Changes:** Energy imbalance can disrupt the normal production of hormones that regulate your menstrual cycle, such as estrogen and progesterone. This can lead to irregular periods, lighter periods, or no periods at all. | **Accurate + Complete**  **Comment:** Provides a clear and thorough explanation of the link between excessive exercise and FHA, including mechanisms (energy imbalance and hormonal disruption) and clinical consequences. Terminology and reasoning are correct, with no missing key concepts. |
| Q1 (Clinician-level) What diagnostic tests should I order if I suspect my patient has FHA?  LLM: ChatGPT-3.5 | If you suspect Functional Hypothalamic Amenorrhea (FHA), the evaluation involves confirming hypothalamic dysfunction and ruling out other causes of secondary amenorrhea. Here's a concise diagnostic guide:  **1. History & Physical Exam**   - **History:** Menstrual irregularities, stress, excessive exercise, disordered eating, weight loss. - **Physical:** Low BMI, malnutrition signs, absence of hyperandrogenism, and other systemic abnormalities.   **2. Key Laboratory Tests**  **To confirm FHA:**   - **FSH, LH, Estradiol:** Low/normal FSH, low LH, low estradiol.   **To exclude other causes:**   - **hCG:** Rule out pregnancy. - **Prolactin:** Rule out hyperprolactinemia. - **TSH, Free T4:** Rule out hypothyroidism. - **Androgens (Testosterone, DHEAS):** Exclude PCOS or androgen-secreting tumors. - **17-Hydroxyprogesterone:** Rule out congenital adrenal hyperplasia.   3. **Imaging (If Needed)**   - **Pelvic Ultrasound**: Thin endometrial stripe, normal ovaries. - **Brain MRI**: If pituitary dysfunction is suspected (e.g., high prolactin).   4. **Functional Tests**   - **Progesterone Challenge Test:** Lack of withdrawal bleeding suggests low estrogen. - **GnRH Stimulation Test**: Attenuated LH response confirms hypothalamic dysfunction (specialized settings).   **5. Additional Considerations**   - **DXA Scan:** Assess bone density due to hypoestrogenism. - **Electrolytes**: Rule out metabolic imbalances.   FHA is diagnosed with evidence of low gonadotropins, hypoestrogenism, and exclusion of other causes, typically linked to stress, undernutrition, or intense exercise. | **Inaccurate**  **Comment:**  While the response lists relevant diagnostic steps, it includes inaccuracies and excessive detail beyond FHA-specific recommendations (e.g., unnecessary tests). The hormonal profile described is incorrect (low/normal FSH and LH rather than typically low levels of both), and some ordering of priorities (e.g., pregnancy test first) is not aligned with current Endocrine Society guidelines. |
| Q7 (Patient-level) What type of doctor should I see if my period has stopped?  LLM: ChatGPT-4 | If your period has stopped, you should consider seeing a gynecologist first. They specialize in women's reproductive health and can help diagnose and treat issues related to menstrual cycles. If the gynecologist deems it necessary, they might also refer you to an endocrinologist, a doctor specializing in hormone-related disorders, to further investigate the cause of your amenorrhea (the absence of menstruation). | **Accurate but incomplete**  **Comment:** Correctly identifies gynecologist and endocrinologist as appropriate specialists but omits guidance on when to seek evaluation, possible red flags, or key steps (e.g., primary care or lifestyle review). Lacks context about causes such as stress, diet, or exercise contributing to FHA. |
